# Multidimensional analysis of matched primary and recurrent glioblastoma identifies contributors to tumor recurrence influencing time to relapse

**DOI:** 10.1093/jnen/nlae108

**Published:** 2024-10-18

**Authors:** Tala Shekarian, Marie-Françoise Ritz, Sabrina Hogan, Tomás A Martins, Philip Schmassmann, Alexandra Gerber, Julien Roux, Deniz Kaymak, Célia Durano, Bettina Burger, Matthias Matter, Gregor Hutter

**Affiliations:** Brain Tumor Immunotherapy and Biology Laboratory, Department of Biomedicine, University of Basel, University Hospital Basel, Basel, Switzerland; Brain Tumor Immunotherapy and Biology Laboratory, Department of Biomedicine, University of Basel, University Hospital Basel, Basel, Switzerland; Brain Tumor Immunotherapy and Biology Laboratory, Department of Biomedicine, University of Basel, University Hospital Basel, Basel, Switzerland; Brain Tumor Immunotherapy and Biology Laboratory, Department of Biomedicine, University of Basel, University Hospital Basel, Basel, Switzerland; Brain Tumor Immunotherapy and Biology Laboratory, Department of Biomedicine, University of Basel, University Hospital Basel, Basel, Switzerland; Brain Tumor Immunotherapy and Biology Laboratory, Department of Biomedicine, University of Basel, University Hospital Basel, Basel, Switzerland; Bioinformatics Core Facility, Department of Biomedicine, University of Basel, Basel, Switzerland; Brain Tumor Immunotherapy and Biology Laboratory, Department of Biomedicine, University of Basel, University Hospital Basel, Basel, Switzerland; Brain Tumor Immunotherapy and Biology Laboratory, Department of Biomedicine, University of Basel, University Hospital Basel, Basel, Switzerland; Dermatology, Department of Biomedicine, University of Basel, Basel, Switzerland; Institute of Pathology, University Hospital Basel, Basel, Switzerland; Brain Tumor Immunotherapy and Biology Laboratory, Department of Biomedicine, University of Basel, University Hospital Basel, Basel, Switzerland; Department of Neurosurgery, University Hospital Basel, Basel, Switzerland

**Keywords:** glioblastoma recurrence, microglia transcriptomic, proteomic, spatial transcriptomic

## Abstract

Glioblastoma (GBM) is a lethal brain tumor without effective treatment options. This study aimed to characterize longitudinal tumor changes in order to find potentially actionable targets to prevent GBM relapse. We extracted RNA and proteins from fresh frozen tumor samples from patient-matched IDH^wt^ WHO grade 4 primary (pGBM) and recurrent (rGBM) tumors for transcriptomics and proteomics analysis. A tissue microarray containing paired tumor samples was processed for spatial transcriptomics analysis. Differentially expressed genes and proteins between pGBM and rGBM were involved in synapse development and myelination. By categorizing patients into short (STTR) and long (LTTR) time-to-lapse, we identified genes/proteins whose expression levels positively or negatively correlated with TTR. In rGBM, expressions of Fcγ receptors (FCGRs) and complement system genes were negatively correlated with TTR, whereas expression of genes involved in DNA methylation was positively correlated with TTR. Spatial transcriptomics of the tumor cells showed enrichment of oligodendrocytes in rGBM. Besides, we observed changes in the myeloid compartment such as a switch from quiescent to activated microglia and an enrichment in B and T cells in rGBM with STTR. Our results uncover a role for activated microglia/macrophages in GBM recurrence and suggest that interfering with these cells may hinder GBM relapse.

## Introduction

Glioblastoma (GBM) is the most aggressive and common malignant brain tumor in adults. Given the widespread tumor cell infiltration into vital or eloquent structures in the brain, complete surgical excision is impossible. Therefore, despite multimodal standard of care (SoC) therapy consisting of maximal resection followed by concurrent chemo-radiation and maintenance adjuvant chemotherapy (temozolomide [TMZ]), the tumor inevitably recurs, with an overall survival of only 15 months.^1^ Thus, GBM is considered an incurable, treatment-resistant cancer, necessitating urgent novel therapeutic strategies. In recurrent GBM (rGBM) emerging after SoC treatment, additional surgery is achievable in only 25% of patients, depending on the location and infiltrative nature of the recurrence.^2^ Subsequently, second-line therapies such as angiogenesis inhibitors or immune checkpoint inhibitors are administered in the frame of experimental clinical trials.^3^ However, all clinical trials failed to display durable remission, prolonged survival, or improved quality of life of the affected patients.^4^

rGBM is often resistant to conventional therapy and is the most aggressive, invasive, and resistant adult brain tumor.^5^ The extreme heterogeneity of rGBM at its cellular, molecular, and genetic levels is more pronounced, making it one of the most complex studied tumors and hindering the identification of potential therapeutic targets.^6^ A study on genetic differences between primary GBM (pGBM) and rGBM has concluded that intratumoral heterogeneity causes the failure of multimodal therapies.^7^

GBM is characterized by a “cold” immune tumor microenvironment (iTME) containing high numbers of suppressive, pro-tumor immune cells such as regulatory T cells (Tregs), tumor-associated microglia and macrophages (TAMs), myeloid-derived suppressor cells (MDSCs), and exhausted T cells.^8^^,^^9^ TAMs are reprogrammed by GBM cells, resulting in an ineffective anti-tumor response. Interactions between TAM and GBM cells promote tumor cell proliferation and migration/invasion, leading to a worse overall prognosis.^10^^,^^11^ Mechanistically, TAMs secrete growth factors, cytokines, and chemokines that remodel the GBM iTME.^12^

Recently, one study investigated the differences between pGBM and rGBM tumor compartments using multi-omic approaches.^13^ However, less is known about the changes in the iTME and the regulation of immunogenicity in rGBM. Therapy-resistant tumor clones give rise to rGBM encompassing a new, heterogeneous iTME with distinct features compared to pGBM.^14^ Changes in the iTME during disease progression and concurrent therapy have been reported, such as decreased numbers of TAMs in rGBM, with an increase of undefined CD45+ immune cell populations.^15^ It is also known that radiotherapy increases the immune responsiveness of tumors by driving immune cell infiltration and enhancing immunogenicity.^16^ This prompted the use of immunotherapy which showed very promising results in a large variety of solid tumors, but not in GBM so far.^17^^,^^18^ Thus, alternative combinatorial therapies targeting rGBM-specific iTME features should be considered.

Molecular markers identified in the primary resected tumors often provide limited information about their subsequent progression, resistance to therapy, or recurrence potential. Therefore, we investigated the differences in gene and protein expression between patient-matched pGBM and rGBM, and correlated the most significant changes in rGBM with time-to-relapse (TTR). Further, we unraveled TTR-specific changes in cell type composition of tumor and myeloid cells using spatial transcriptomics. Our results contribute to expand the understanding of the underlying biological processes that lead to GBM recurrence and underscore potentially targetable pathways for treating rGBM.

## Methods

### Ethics approval and consent to participate

All patients provided written, informed consent according to the legislation of the local ethical committee (EKNZ 02019-02358), for material and data collection supporting these analyses. The study was conducted following the ethical principles of the Declaration of Helsinki, regulatory requirements, and the code of Good Clinical Practice.

### Demographic and clinical information of the patient cohort

We retrospectively included 18 patients with pGBM and rGBM (WHO grade 4, *IDH* wild type) operated at the Neurosurgery Clinic, University Hospital of Basel from 2010 to 2020. GBM was diagnosed based on typical magnetic resonance imaging (MRI) characteristics and confirmed postoperatively by board-certified neuropathologists (Institute of Pathology and Genetics, University Hospital Basel, Switzerland) using complementary histological and molecular techniques. This diagnosis was also retrospectively confirmed by copy number variation and methylation-based tumor classification. Twelve patients were tested negative for MGMT promoter methylation, whereas 5 patients exhibited complete or partial MGMT promoter methylation, and methylation status was unknown for 1 patient (Table 1). All subjects received perioperative corticosteroids followed by conventional chemo- and radiotherapy before the second surgery. Some patients received adjuvant therapy such as Avastin, Lomustin, Fortemustin, or Optune after the second surgery. Time to relapse (TTR) was determined by calculating the interval between the dates of first and second surgeries (range 148-921 days). Patients’ information, including age, gender, treatments, and clinical data (OS and TTR) are summarized in Table 1 and Table S1.

**Table 1.** Compiled patient information dichotomized according to times to relapse.

	Relapse time (cut-off = 309 d)	
Characteristic	Long, *n* = 9^a^	Short, *n* = 9^a^
Age at diagnosis	57 (8)	57 (9)
Sex		
Female	2 (22%)	2 (22%)
Male	7 (78%)	7 (78%)
Diagnosis		
GBM, WHO grade 4	9 (100%)	9 (100%)
IDH status		
Wild-type	9 (100%)	9 (100%)
MGMT-promotor methylation status		
Negative	6 (75%)	6 (67%)
Positive	2 (25%)	3 (33%)
NA	1	0
Adjuvant treatment		
CRT	9 (100%)	9 (100%)
Time to relapse (d)	471 (208)	210 (32)
Survival since first surgery (d)	801 (317)	457 (167)
Subtype		
Classical	1 (11%)	3 (33%)
Mesenchymal	1 (11%)	1 (11%)
Proneural	7 (78%)	5 (56%)
Transcriptomic analysis	8	8
Proteomics analysis	2	4
GeoMX spatial transcriptomic analysis	7	3

a Mean (SD); d, days; *n* (%), *n*; CRT, chemo-radiotherapy; NA, not available.

### Total RNA extraction and gene expression assay

GBM tissue samples from 17 patients were collected directly in the operating theater during primary (first) and recurrent (second) surgeries and immediately stored in RNAlater stabilization solution (Thermo Fisher Scientific, Waltham, MA, USA) and stored at −80 °C. For RNA extraction, tumors were mechanically dissociated in lysis buffer and total RNA was extracted using the AllPrep DNA/RNA/protein mini Kit (Qiagen, USA, Cat. No. 80004) according to the manufacturer’s protocol. RNA purity and concentration were assessed using the 2100 Bioanalyzer (Agilent Technologies, Santa Clara, CA, USA). Gene expression was analyzed using the nCounter PanCancer IO 360 and the Neuroinflammation panels comprising 770 genes each (NanoString Technologies, Seattle, WA, USA), using 50 ng of total RNA per sample, according to the manufacturer’s protocol.

### Transcriptomic data analysis

Data collection was performed using the nSolver Analysis system and nCounter Advanced Analysis 2.0 software (Nanostring Technologies). Normalization was performed to account for differences in sample composition and to correct for technical variability. Given the large number of genes in each panel we chose to use a classical normalization approach; we utilized the *calcNormFactors* function from the edgeR package to compute normalization factors for both panels independently. The expression values were further normalized using the *normalizeBetweenArrays* function, applying the cyclic loess method. This method is effective in adjusting for systematic differences between arrays. Importantly, we down-weighted the control probes (both negative and positive) to zero to exclude them from influencing the normalization process. Post-normalization, the data from the two panels were re-aggregated to form a comprehensive dataset for analysis. Samples that passed the normalization steps were included for subsequent statistical analysis. The final dataset contains 30 samples (primary and recurrent) from 15 patients and 1306 unique genes. A comparison of gene expression levels between pGBM and rGBM was performed using the *limma* package from the Bioconductor. The analysis included a linear model where the patient effect was explicitly modelled to account for inter-patient variability. The same model was used to evaluate differences between patient samples categorized in short and long TTR, with a threshold set at 309 days to create balanced groups in terms of sample size.

The Benjamini-Hochberg correction method was applied to adjust *P*-values for multiple testing, ensuring control over the false discovery rate. Hierarchical clusterings of gene expression data was conducted and adjusted for patient effect using the *removeBatchEffect* function from the *limma* package. This adjustment helps to mitigate batch effects and other non-biological variations that could confound the results. For functional enrichment analysis, gene set enrichment analysis and over-representation analysis of GO terms were performed using the *clusterProfiler* package. The Benjamini-Hochberg procedure was employed again to calculate adjusted p-values and identify significantly enriched GO terms associated with the differentially expressed genes.

### Proteomic and data analysis

Paired fresh-frozen tissue samples were available from a subset of 6 patients. Following histological evaluation of cryosections stained with hematoxylin to exclude tissue with excessive hemorrhage or necrosis, proteins were extracted using the standard protocol of the Protein Core Facility, Biocenter, University of Basel, and processed for proteomic analysis. Further details and experimental parameters are provided in Methods S1.

Comparison between pGBM and rGBM expression levels was performed using the free open-source R package *limma* from Bioconductor, for the analysis of gene differential expression. with patient effect included in the model to account for inter-patient variability and ensure accurate differential expression analysis. The Benjamini-Hochberg correction was applied to obtain adjusted p-values, controlling the false discovery rate and providing more reliable significance levels. Hierarchical clusterings of gene expression data were adjusted for patient effect using the *removeBatchEffect* function from the *limma* package, effectively removing batch effects and enhancing the clarity of clustering results. The gene set enrichment analysis and over-representation analysis of GO terms were performed using the *clusterProfiler* package and the adjusted *P*-values were calculated using the Benjamini-Hochberg procedure, ensuring the robustness of the enrichment results by accounting for multiple comparisons.

### Spatial transcriptomics

A tissue microarray of a subset of 7 patient-paired primary and recurrent FFPE GBM samples was assembled (overview shown in Figure S3) and subjected to spatial transcriptomic analysis using the NanoString’s digital spatial profiling technology (GeoMx DSP, NanoString, Seattle, WA, USA) as detailed in Methods S2.

### GeoMx RNA data processing

GeoMx DSP data were evaluated and prepared for downstream analysis in R, following the GeoMx-NGS gene expression analysis workflow until the filtering step. The workflow included initial quality control checks to ensure data integrity and accuracy. Segments with less than 4% of the genes detected were removed to eliminate low-quality data and reduce noise in the downstream analysis. The cut-off for gene detection was set at 2%, ensuring that only reliably detected genes were included in the analysis. Reads from duplicated samples were summed to enhance the robustness of the data and improve the reliability of the results.

The package *limma* was used to perform between-samples cyclic loess normalization and to test for differential expression using a linear model (lmFit). Cyclic loess normalization was chosen to address systematic biases and ensure accurate comparison of expression levels across samples. The differential expression analysis was conducted by fitting a linear model to the normalized data, allowing for the identification of genes with statistically significant changes in expression between conditions.

Principal component analysis (PCA) was performed on the filtered gene set using log2 cpm (count per million) values and was plotted using the fviz_pca function from the factoextra R package. PCA was utilized to reduce the dimensionality of the dataset and to visualize the overall structure of the data, highlighting the main sources of variation. SpatialDecon (v1.4.3) was used to determine the cell type composition within the samples. This involved deconvoluting the spatial transcriptomics data to estimate the proportion of different cell types present in each sample, providing insights into the cellular heterogeneity The negative probes were used to estimate each data point’s expected background. The Darmanis Brain Atlas cell profile matrix was downloaded from the SpatialDecon library and used to deconvolute the GFAP+ population. A custom cell profile matrix was constructed using scRNA sequencing data from Schmassmann et al..^19^ Cell populations of interest were selected in the profile matrices. The spatialdecon function was run using nuclei count to estimate the total number of cells.

### Data and material availability

All data needed to evaluate the conclusions in the article are present in the article and/or the Supplementary Materials. Transcriptomic and spatial transcriptomic data have been deposited on the GEO repository under the reference numbers GSE254875. Proteomic data has been deposited to ProteomeXchange under the reference PXD041647.

## Results

**Figure 1.**
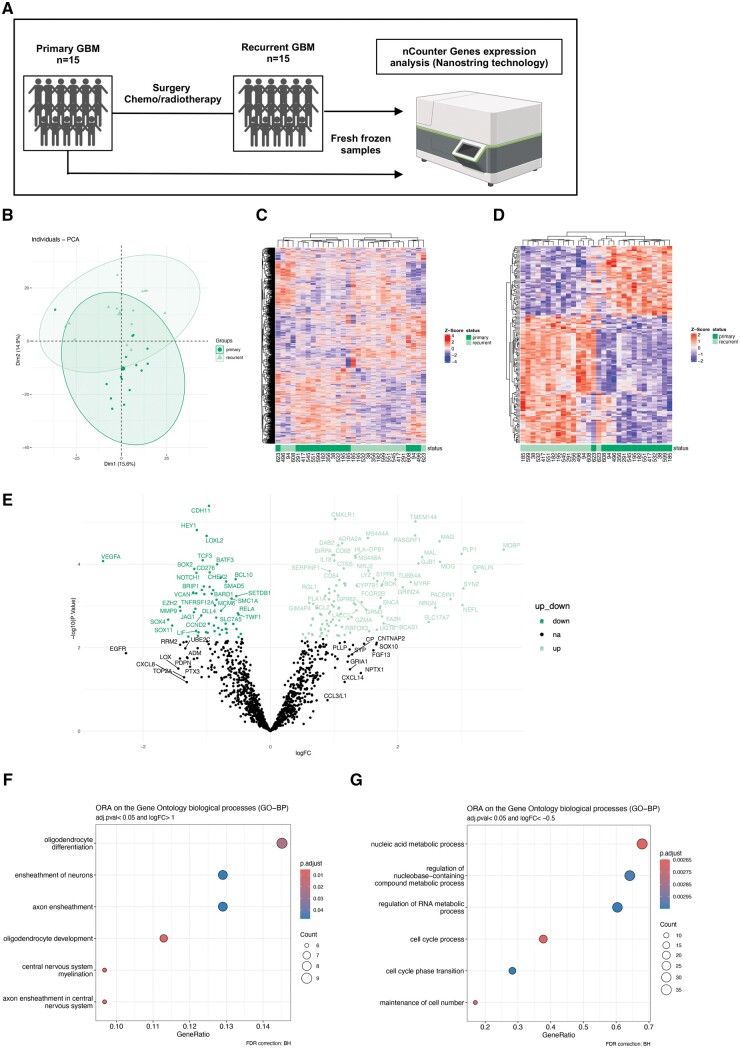
Targeted transcriptomic analysis of patient-matched pGBM and rGBM reveals synapse formation and oligodendrocyte differentiation as pathways activated in rGBM. (A) Schematic experimental setup for gene expression analysis using RNA extracted from fresh-frozen samples from 15 patient-matched pGBM and rGBM tumors using Nanostring nCounter system. (B) PCA plot of all samples included in the analysis, each dot represents a patient, color-coded according to pGBM or rGBM. Dim1 represents PC1 and Dim2 PC2. The percentage of variation explained by each component is shown in parentheses. (C) Hierarchical clustering heatmap of the genes expressed across all samples. *Z*-score transformation was performed for each protein. Genes with similar expression patterns cluster together. Relative expressions were scaled from red (high expression) to blue (low expression). Each column represents a sample (dark green: pGBM, light green: rGBM), and each row represents a gene. (D) Hierarchical clustering heatmap of the differentially expressed genes across all samples. (E) Volcano plot showing significantly differentially overexpressed genes in rGBM (light green) and pGBM (dark green) (adjusted *P* < .05, log_2_FC > 0 and log_2_FC < 0). (F, G) Dot plots of the enriched biological processes (GO-BP) determined by ORA using overexpressed genes in rGBM and pGBM, respectively.

We explored the transcriptional immune signatures of 15 patient-matched pGBM and rGBM specimens (age range of 39-65 years at the time of diagnosis) by performing a targeted analysis of 1320 genes involved in the iTME and the tumor, using two specific transcriptomic panels (Figure 1A). The 2 panels (“ISO360 pathways” and “Neuroinflammation pathways”) cover genes involved in (1) the tumor signature (immunogenicity, sensitivity, inhibitory mechanisms), the tumor microenvironment (stromal factors, metabolism), and the immune responses (immune cell abundance, anti-tumor activity/signaling), and (2) the neuroinflammation signature, targeting genes from CNS-specific cells (neurons, astrocytes, microglia, oligodendrocytes), endothelial cells and peripheral immune cells (B and T cells, T reg, Th1 cells, dendritic cells, macrophages, microglia, neutrophils, mast cells, CD45-positive cells). The combination of these 2 panels thus covers a broad variety of genes that are included, implicated in the immune system, nervous system and stroma.

### Genes associated with myelin formation are enriched in rGBM

Principal component analysis (PCA) of all investigated genes showed partial segregation of pGBM and rGBM (Figure 1B). Unsupervised hierarchical clustering reinforced these results by consistently separating pGBM from rGBM (Figure 1C), suggesting distinct signature profiles between the two groups. About 167 differentially expressed genes (DEGs) were identified between pGBM and rGBM (Figure 1D and Table S2). Significantly upregulated DEGs in rGBM encoded for myelin-associated proteins/glycoproteins, such as *MAG*, *MAL*, *MOBP*, *MOG*, and *PLP1*. In contrast, downregulated DEGs in rGBM encoded for proteins involved in angiogenesis (like *VEGFA*), in extracellular matrix formation (like *CDH11* and *LOXL2*) or associated to reduced stemness markers (like *NOTCH1*, *HEY1*, and *SOX2*).

The significantly upregulated DEGs in rGBM samples, shown in Figure 1E (light green), were subjected to Over-Representation Analysis (ORA) of gene ontology classification for biological processes (GO-BP) (Figure 1F). These DEGs were enriched in the terms “ensheathment of neurons,” “central nervous system myelination,” and “oligodendrocyte differentiation and development” suggesting higher myelination in rGBM compared to pGBM. According to the GO-BP enrichment analysis, the DEGs upregulated in pGBM (dark green in Figure 1E) are related to “cell cycle process” suggesting more proliferative characteristics of tumor cells in pGBM compared to its recurrence (Figure 1G).

Collectively, these targeted transcriptomic results indicate that despite the vast inter- and intratumoral heterogeneity observed in GBM, rGBM and pGBM transcriptomic profiles differ in their signatures, suggestive of an enhanced myelination and oligodendrocyte differentiation at recurrence.

### Proteomic analysis highlights synaptic signaling as a major feature enriched in rGBM

**Figure 2.**
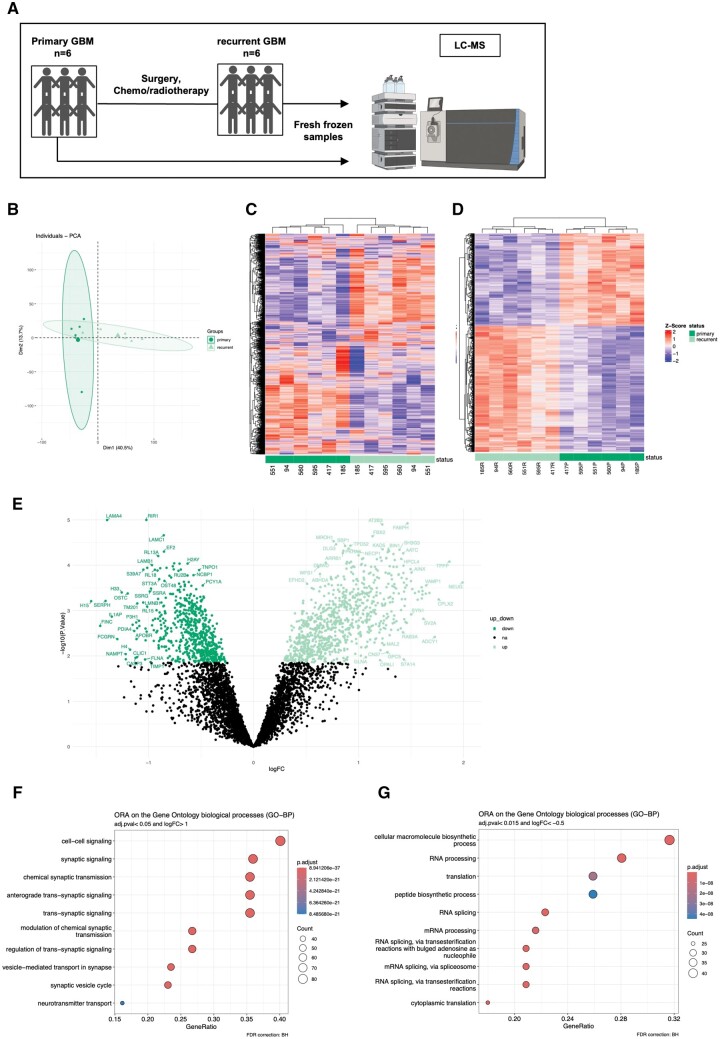
Unbiased LC-MS based proteomic confirms synapse signaling as a major pathway in rGBM. (A) Experimental setup for LC-MS based proteomic analysis of 6 fresh frozen patient-matched pGBM and rGBM samples (clinical parameters highlighted in Table 1 and Table S1). (B) PCA plot of all samples included in the analysis, each dot represents a patient, color coded according to pGBM or rGBM. Dim1 represents PC1 and Dim2 PC2. The percentage of variation explained by each component is shown in parentheses. (C) Hierarchical clustering heatmap of all the identified proteins across all samples. Z-score transformation was performed for each protein. Proteins with similar expression patterns were clustered together. Relative expressions were scaled from red (high expression) to blue (low expression). Each column represents a sample (dark green: pGBM, light green: rGBM), and each row represents a protein. (D) Hierarchical clustering heatmap of all DEPs across all samples. (E) Volcano plots showing significantly differentially overexpressed proteins in rGBM (light green) and pGBM (dark green) (adjusted *P* < .05, log_2_FC > 0 and log_2_FC < 0). (F, G) Dot plots of the enriched biological processes (GO-BP) determined by ORA using overexpressed proteins in rGBM and pGBM, respectively.

To strengthen our findings, we performed LC-MS-based proteomics on a subset of 6 patient-matched tumors (Figure 2A), identifying 8932 proteins (5214 complete cases). Amongst them, 1539 were differently expressed between pGBM and rGBM, with a false discovery rate (FDR)-adjusted *P*-value <.05. PCA effectively differentiated pGBM from rGBM samples, revealing a distinct clustering pattern between the 2 groups, except for 1 rGBM sample, co-segregating with pGBM samples (Figure 2B). Consistently, hierarchical clustering of all assessed proteins (Figure 2C), and differentially expressed proteins (DEPs, Figure 2D) grouped rGBM samples separately from pGBM samples. Pathway analysis revealed that among the 883 significantly upregulated DEPs in rGBM (showed in light green in Figure 2E, listed in Table S2), enrichment was observed in pathways related to “synaptic signaling” and “vesicle-mediated transport in synapse” (Figure 2F). The top DEPs associated with these features comprised NEUG, FABPH, CPLX2, ADCY1, and SYPH.

Pathway analysis of the 656 significantly upregulated DEPs in pGBM (showed in dark green in Figure 2E) revealed enrichment of proteins involved in “mRNA metabolic processes” and “RNA processing,” such as SMD3, U2AF1 and FBRL (Figure 2G). We performed a comparative analysis between transcriptomic and proteomic datasets, and we found a good correlation (*R* = 0.41, *P* < 2.2e^−16^) between the genes and proteins differently expressed in our paired samples (*n* = 8).

### High expression of FCGRs and complement component genes is associated with STTR in rGBM

**Figure 3.**
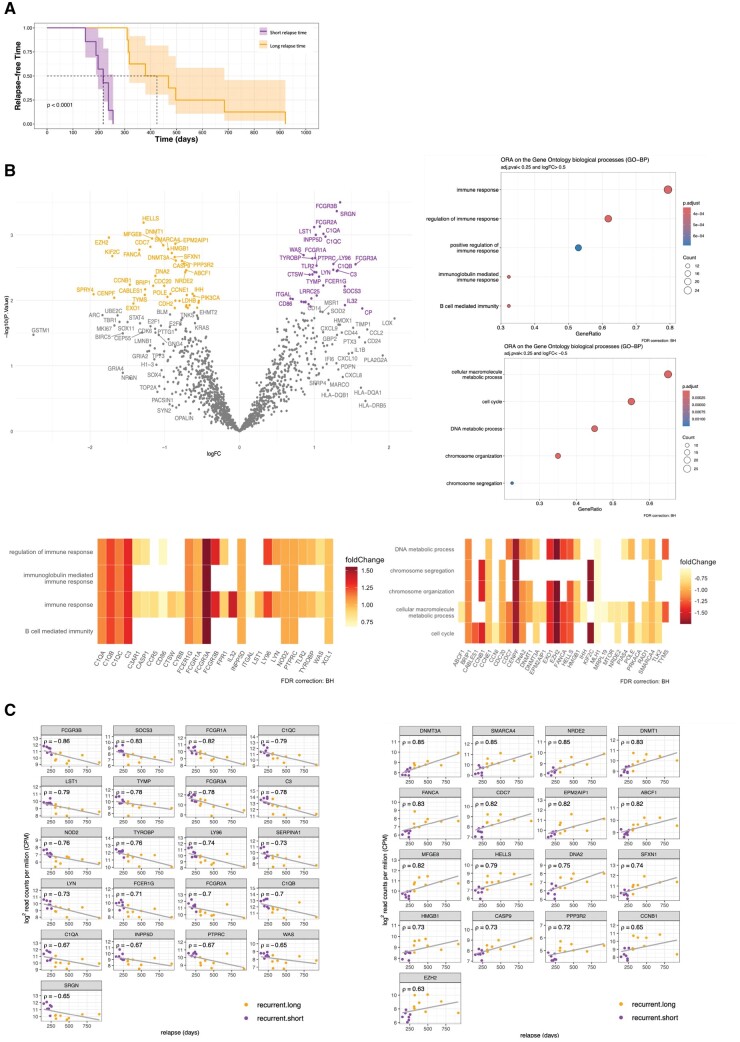
Expressions of immunoglobulin binding receptors and complement system components are higher in the subset of patients with STTR and inversely correlate with TTR. (A) Kaplan-Meier Estimates of time to relapse in rGBM for patients with STTR (<309 days) and LTTR (≥309 days). (B) Volcano plot showing DEGs between patients with STTR vs LTTR in rGBM samples. Genes overrepresented in rGBM with STTR (purple) or LTTR (orange) are highlighted (adj. *P* < .25, logFC > 0.5 and logFC < −0.5), dot-plots of the corresponding enriched biological processes (GO-BP) determined by ORA (right part) and expression of genes enriched in at least one of the 4 most enriched processes (lower part). (C) Scatterplots showing expression of the top negatively (*r* < −0.63, left) or positively (*r* ≥ 0.63, right) correlated gene with TTR in rGBM samples.

To investigate mechanisms underlying recurrence, we focused on the DEGs upregulated in rGBM. We stratified rGBM samples into 2 groups based on patients’ TTR: short TTR ([STTR] < 309 days) and long TTR ([LTTR] ≥ 309 days) (Figure 3A). We identified 75 interesting DEGs between both groups (*P* < .05, Figure 3B, upper part left, listed in Table S2). GO-ORA analysis conducted on DEGs overexpressed in STTR revealed enrichment in “regulation of immune response” and “B cell-mediated immunity.” Conversely, DEGs overexpressed in LTTR were specifically enriched in “cell cycle” and “DNA metabolic process” pathways (Figure 3B, upper part right). The DEGs present in the most enriched terms (Figure 3B, lower part), comprise genes coding for the complement system (*C1QA*, *C1QB*, *C1QC*, *C3*, and *C3AR1*) and FCG receptors (*FCGR1A* (also known as *CD64*), *FCGR2A*, *FCGR3A*, *FCGR3B*) which are upregulated in samples with STTR, and *DNMT3A* and *DNMT1*, *SMARCA4*, *MLH1*, and *TLK2*, which are highly expressed in samples with LTTR.

Analysis of the correlation between expression levels and TTR identified DEGs that are overexpressed in STTR and strongly negatively associated with TTR, suggesting an unfavorable impact on patients’ overall outcome (Figure 3C left, and Figure S1A). On the other hand genes correlating positively with TTR in rGBM (Figure 3C right, and Figure S1B) may confer a survival benefit for patients. To validate our findings, we compared our data with selected data from the GLASS consortium, established in order to identify the drivers of treatment resistance in glioma. We included primary and recurrent IDH-wild type GBM tumors, for which TTR information was available. When comparing gene expression levels with TTR for the top genes identified in the present study, we saw a comparable positive or negative correlation with TTR (Figure S1C and D, respectively), with the negative correlations reaching statistical significance, confirming our observations with the larger GLASS cohort.

### Among DEPs, expression of FCGR1 (CD64) and SHIP1 correlated with STTR

We next correlated the expression levels of the proteins corresponding to the top DEGs in rGBM with TTR (Figure S2A). Despite the small number of samples (4 with STTR, and 2 with LTTR), significant negative correlations for CD64 and SHIP1 expressions with TTR were observed. Some proteins displayed higher expressions in LTTR rGBM, however, none of them showed correlations with TTR (Figure S2B).

### Spatial transcriptomic identified cell type composition differences in the CD64^+^ myeloid population but not in GFAP^+^ cells during disease progression

**Figure 4.**
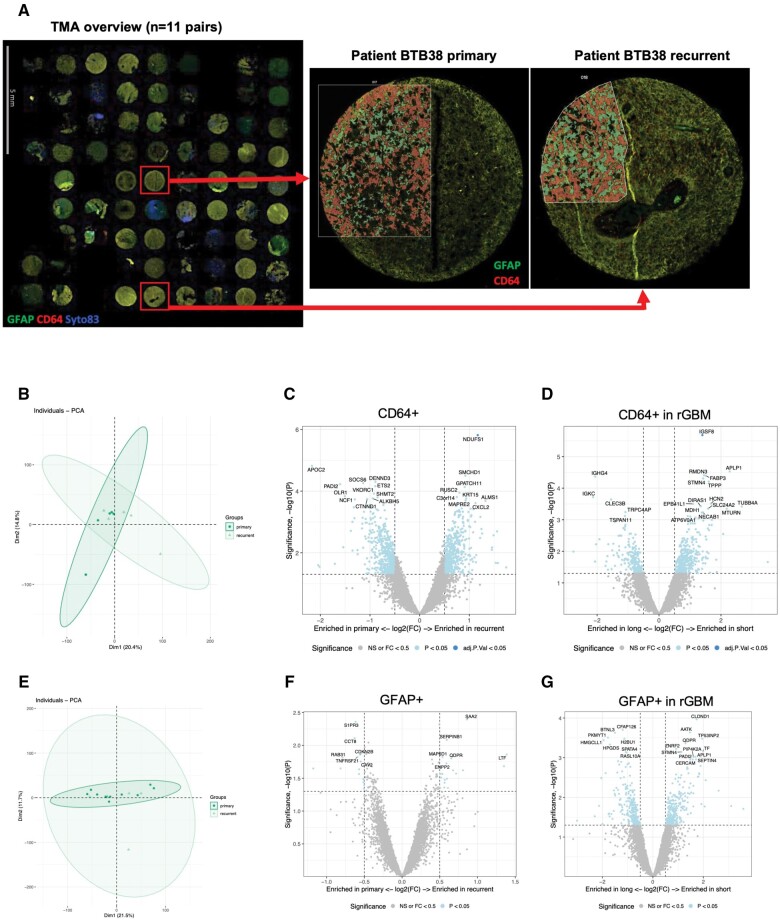
Spatial transcriptomic of CD64- and GFAP-expressing cell populations revealed subtle changes in microglia subtypes in rGBM. (A) Representative tissue microarray section containing 7 pairs of patient-matched pGBM and rGBM samples stained for GFAP (red), CD64 (green), and DAPI (cell nuclei in blue) identifying ROIs for GFAP+, CD64+ cellular fractions and nuclei, respectively, and selected for whole transcriptome sequencing. Inserts show magnifications of ROIs from pGBM and rGBM from patient BTB38 as an example. (B) PCA plot for CD64+ samples in pGBM and rGBM. Dim1 represents PC1 and Dim2 PC2. The percentage of variation explained by each component is shown in parentheses. (C) Volcano plot showing DEG between pGBM and rGBM in CD64+ cells. (D) Volcano plot showing DEG between STTR and LTTR in CD641 rGBM cells. (E) PCA plot for GFAP+ samples in pGBM and rGBM. Dim1 represents PC1 and Dim2 PC2. The percentage of variation explained by each component is shown in parentheses. (F) Volcano plot showing DEG between pGBM and rGBM in GFAP+ cells. (G) Volcano plot showing DEG between STTR and LTTR in GFAP+ rGBM cells. For all the volcano plots *P* < .05 in light blue, adj. *P* < .05 in dark blue, vertical dashed lines at logFC > 0.5 or < −0.5, horizontal dashed line at *P* < .05.

To focus on changes occurring specifically in myeloid and neoplastic cells, we performed spatial transcriptomics analysis on GFAP^+^ cells (tumor cells, astrocytes) and CD64^+^ cells to gain a more granular understanding of myeloid/tumor transcriptional state changes on the path from pGBM to rGBM. Since CD64 expression appeared to be highly correlated with TTR at the gene and protein levels, we chose this marker for specifically capture the changes in myeloid cells. A cohort of 11 patient-paired pGBM and rGBM tissue cores were assembled on a tissue microarray. Although fluorescent cells were intimately intertwined in the tumor tissue, both fluorescent signals could be well distinguished, allowing the capture of both populations (Figure 4A). The quality/quantity of the sequencing data was, however, below the standard limit for a large number of regions of interest (ROIs). Consequently, only 3 matched pairs, 2 primary samples, and 2 recurrent samples could be fully exploited for the CD64^+^ ROIs. For the GFAP^+^ ROIs, 5 matched pairs and 5 primary samples were included.

PCA of the CD64^+^ population did not show clear segregation between pGBM and rGBM tumors (Figure 4B). We identified several DEGs among CD64^+^ cells in pGBM vs rGBM; however, none reached statistical significance (Figure 4C, *P* < .05, but adj. *P* > .05, listed in Table S2). When comparing gene expression between rGBM with STTR and LTTR, *IGSF8*, which codes for an immune checkpoint, was significantly overexpressed in the CD64^+^ population with STTR (Figure 4D). As the PCA of GFAP^+^ ROIs already suggested (Figure 4E), gene expression analysis failed to uncover significant DEGs between pGBM and rGBM (Figure 4F, complete list in Table S2) and between samples with LTTR and STTR in rGBM (Figure 4G, Table S2).

**Figure 5.**
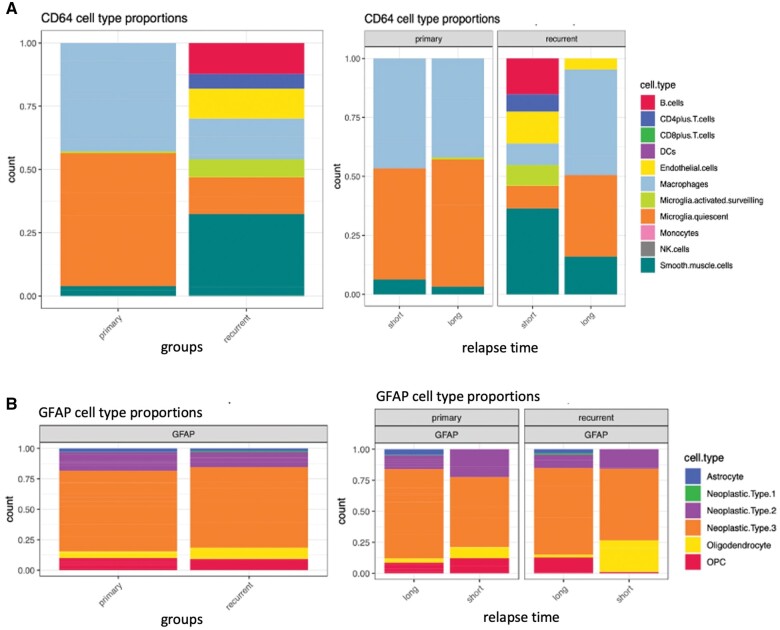
Cellular deconvolution of CD64+ and GFAP+ cell populations revealed subtle changes in microglia subtypes in rGBM. (A) Relative cell type abundance in the CD64+ population, divided into pGBM and rGBM samples (left chart) and grouped according to short or long TTR (right chart). (B) Relative cell type abundance in the GFAP+ population was divided into pGBM and rGBM samples (left chart) and grouped according to short or long TTR (right chart).

Since both markers used for spatial transcriptomics are expressed by various cell types, we attempted to detect composition changes in specific cell types using cellular deconvolution. Among the CD64^+^ population, we identified mainly quiescent microglia, macrophages and smooth muscle cells in pGBM (Figure 5A). The most prominent changes in cell type composition upon recurrence were observed in 2 recurrent samples: quiescent microglia were reduced whereas activated microglia appeared in rGBM with STTR, together with a small proportion of B and T cells. Macrophages were equally present in proportion in pGBM and rGBM during disease progression. An increase in the proportion of endothelial cells and smooth muscle cells in rGBM samples was also observed (Figure 5B and Figure S5).

The GFAP-expressing cohort consisted of different cell types, as identified using the cell profile matrix of Darmanis et al.^10^ Since GBM is a tumor of glial origin, GFAP was chosen in order to identify neoplastic cells, normal astrocytes, oligodendrocytes and Oligodendrocyte precursor cells (OPCs) known to express this protein. We observed that most of the GFAP+ cells were classified as neoplastic cells (Figure 5C and D), with very low proportion of normal cells, illustrating the high content of neoplastic cells in our samples. When comparing the relative proportion of these various cell types in pGBM and rGBM, only minor differences were observed, such as a slight increase in the proportion of oligodendrocytes in rGBM samples with STTR (Figure 5D and Figure S4B).

## Discussion

In this study, protein and RNA from patient-matched pGBM and rGBM samples were analyzed to investigate mechanisms involved in tumor relapse. Our results confirmed several previous observations, such as the decrease in the cell cycle pathway in rGBM as seen in bulk RNA-seq and proteomics of a larger cohort^20^ and in a proteogenomic analysis demonstrating that GBM evolution under therapy is accompanied by the collapse of the cell cycle, mitosis, and DNA damage/repair activities sustained by the loss of the genetic alterations and reduced abundance of the proteins involved in these functions.^21^

### Myelination and neuronal connectivity processes are activated in rGBM

In the transcriptomic analysis, we identified an overexpression of genes associated with processes that govern the formation and maintenance of myelin sheaths around axons, which are critical for neural signal transmission and neural connectivity, in rGBM. The processes of ensheathment and myelination, typically associated with protecting and insulating neurons, might contribute to creating a more permissive environment for tumor cell invasion and spread.

Recurrent GBM is often characterized by a shift towards stem-cell-like or progenitor-like cells like OPCs. OPCs, which are responsible for the maintenance and regeneration of myelin, rapidly migrate to injured sites, reaching regions of traumatic brain injury within 1 day.^22^ This may participate in the development of rGBM, by causing GBM cells to acquire stem cell-like properties and chemo-radioresistance.^23^ Our results suggest that the cellular characteristics of GBM cells which resemble various stages of oligodendrocyte differentiation^24^ may become more pronounced during tumor recurrence. Moreover, our proteomics analysis highlighted the activation of processes related to synaptic function and signaling in rGBM. Earlier studies,^25^ as well as a publication from the GLASS consortium,^26^ have described the enrichment of synapse signaling and myelination pathways in rGBM, suggesting that regenerative processes in the CNS at the site of recurrence may facilitate the integration of tumor cells into the TME. Moreover, neurons have been identified as crucial components of the TME, influencing malignant growth in an activity-dependent manner.^25^ The synaptic integration of GBM tumor cells is now considered a major driver of tumor progression^25^^,^  ^27^^,^^28^ that negatively impacts cognition and survival.^29^ A recent integrative proteogenomic analysis of 123 longitudinal GBM pairs identified a highly proliferative cellular state at diagnosis, which is replaced by the activation of neuronal transition and synaptogenic pathways in recurrent tumors.^21^ This study demonstrated that neurogenesis and synapse formation at recurrence are accompanied by post-translational activation of the wingless-related integration site (WNT)/planar cell polarity (PCP) signaling pathway and BRAF protein kinase. Overall, the processes highlighted in our analysis collectively suggest an improved adaptation and survival of GBM cells, potentially facilitating tumor recurrence and progression.

### FCGRs, complement components, and inflammatory mediators influence the progression of rGBM

We focused on DEGs and DEPs that showed a strong correlation between their expression levels and TTR, aiming to identify potential targetable markers of rapidly relapsing tumors. Recurrent tumors that evaded SoC treatment may *de novo* express therapy resistance genes that are inversely correlated with TTR. Indeed, we identified 21 genes that were strongly negatively correlated with TTR in our study. Some of these genes, such as FCGR1A (CD64), have been identified in previous studies as having prognostic value for GBM radioresistance.^30^ FCGR1A, a high-affinity FCGR expressed on the surface of myelomonocytic and dendritic cells, may serve as a prognostic marker associated with immune infiltration levels across various cancers. Human microglia express FCGR1, FCGR2A, FCGR2B, and FCGR3A, albeit at very low levels under normal conditions. Their expression on microglia is increased in the CNS of patients with neuroinflammatory diseases such as Alzheimer’s disease,^31^ in which the FGCRs play a role in plaque clearance. In GBM, TAMs are considered important drivers of the local immunosuppressive TME, playing a significant role in tumor progression and resistance to immunomodulating therapies.^32^ In our study, TAMs expressing high levels of FCGRs such as FCGR1 and FCGR3A in rGBM were identified as activated microglia and are associated with STTR. Proteomics analysis also identified SHIP1 as a protein negatively correlated with TTR. SHIP1 is expressed on immune cells such as B and T cells, NK cells, dendritic cells, mast cells, and macrophages. In macrophages, reduced SHIP1 expression promotes M2 macrophage development,^33^ suggesting that high expression of SHIP1 may favor the M1 pro-tumoral phenotype in rGBM.

Complement components C1qA, C1qB, and C1qC were highly expressed in rGBM and inversely correlated with TTR. In the CNS, proteins from the complement system are constrained to microglia, oligodendrocytes, astrocytes, and ependymal cells.^34^ C1q may serve as a reliable marker of microglial activation ranging from undetectable levels of C1q biosynthesis in resting microglia, to abundant C1q expression in activated, non-ramified microglia.^35^ In GBM, the presence of C1q is highly concentrated in TAM and necrotic debris,^36^ thereby promoting immunosuppression and glioma cell proliferation. Other genes identified in our study may influence microglia signatures, such as *TYROBP*, coding for the tyrosine kinase binding protein DAP12. The latter has been shown to switch microglia states from homeostatic to disease-associated, activated microglia in Alzheimer's disease.^37^  *LST1*, mainly expressed in degranulating macrophages, was also strongly associated with STTR. Abnormal expression of *SOCS1* and *SOCS3* in microglia and astrocytes is associated with poor prognosis in GBM.^38^ Moreover, the activation of the JAK/STAT3/SOCS3 signaling pathway can promote the formation of an inflammatory microenvironment in GBM. SOCS proteins also play a role in the epigenetic regulation of GBM cells through methylation and contribute to chemotherapy resistance in GBM.^39^ Interrogating the proteomics results, several proteins encoded by these genes were also associated with TTR, including proteins from the C1q family, FCGR1/FCGRB, FCGEG, and PTPRC.

### DNA methylation and other genes favoring LTTR

Genes with higher expression in LTTR rGBM may be associated with a less aggressive phenotype. Higher expression of DNA methyltransferases *DNMT1*, *DNMT3A*, and *SMARCA4* were observed in our LTTR patients. Indeed, DNA methylation is associated with TMZ sensitivity in glioma cells.^40^ These observations support a favorable role of DNA methyltransferases in fine-tuning chemoresistance after GBM SoC treatment.

In addition, expression of *MFGE8*, *SFXN1*, and *ABCF1* positively correlated with TTR in rGBM. *MFGE8* promotes phagocytic removal of apoptotic cells leading to tolerogenic immune responses, and vascular endothelial growth factor (VEGF)-induced angiogenesis.^41^ This phagocytic function may help delay tumor relapse. *SFXN1* affects mitochondrial function and iron transport and may also modulate tumor progression in gliomas, thus influencing survival.^42^  *ABCF1*, a multidrug resistance gene, confers resistance of cancer cells to a range of anti-cancer agents.^43^ There is also evidence of its action to promote cell growth and metastasis in GBM.^44^ These genes, through their respective roles, may contribute to creating a less favorable environment for rapid tumor growth and recurrence. Their positive correlation with LTTR in rGBM suggests that higher expression or functional activity of these genes might be associated with slower disease progression and extended periods before relapse.

### Changes in neoplastic and myeloid cell populations influencing disease progression

Dissecting the heterogeneity of the cell types present in pGBM and rGBM may be of critical importance to target-specific cell populations in a clinical setting. Using spatial transcriptomics, we explored the transcriptomic changes taking place, specifically in the GFAP+ neoplastic cell population and CD64^+^ myeloid cells, during disease recurrence.

Gene expressions in GFAP^+^ cells, composed mainly of cancer cells, were not significantly changed after SoC therapies. Our study did not highlight specific gene expression changes in GFAP^+^ tumor cells that significantly influenced TTR. In addition, no significant changes in the cell type composition within the GFAP^+^ population were observed between pGBM and rGBM, except an increase in oligodendrocytes, confirming the observation of enhanced myelination in rGBM.

Concerning the CD64^+^ myeloid population, no significant differences in gene expression were found between pGBM and rGBM. When correlating gene expression in rGBM samples with TTR, only one gene, *IGSF8*, showed significantly elevated levels in samples with STTR. Interestingly, IGSF8 has recently been identified as an immune checkpoint that suppresses natural killer (NK) cell function in tumors.^45^ Its overexpression is associated with low immune infiltration and worse clinical outcomes in many cancers.

The change in the microglial abundance toward activated microglia in rGBM indicates that CD64^+^, M1-like microglia, may contribute to radio- and chemoresistance and lead to GBM recurrence. Therefore, CD64^+^ microglia in the TME represent definitive therapeutic targets for the development of new drugs.

Macrophages are also known to express a range of receptors such as Fc receptors, including CD64, which are upregulated under pro-inflammatory conditions. This cell population was unchanged in proportion between pGBM and rGBM and therefore, may not influence the progression of the disease.

Some cells in the CD64^+^ population also showed expression of B cell markers in 2 out of 5 rGBM samples. Interestingly, B cells have been reported to be part of the GBM immune landscape, characterized by an immunosuppressive activity toward activated CD8^+^ T cells, through overexpression of inhibitory molecules such as PD-L1 and CD155, and production of the immunosuppressive cytokines TGFβ and IL10.^46^

We noted an increase in the proportion of endothelial cells in the CD64^+^ cell population in rGBM samples. Since endothelial cells are not known to express CD64, this might result from contamination by blood vessels isolated together with the CD64^+^ cell population, due to the strong adhesion of CD64+ macrophages to vascular structures.^47^

This study has several limitations; the small cohort of patient-paired GBM samples and the small tumor fractions studied reduce the confidence of the findings. Moreover, segregating the samples according to TTR could lower the chance of detecting a real difference between STTR and LTTR samples. However, despite the small number of paired samples, we could validate previously reported observations, such as decreased cell cycle and increased neurogenesis and synaptic formation in rGBM. Additionally, we found strong correlations between gene/protein expressions and TTR, especially for increased DNA methylation in LTTR samples. Although correlation does not imply causation, it highlights a relationship between both factors. Therefore, it would be essential to validate these associations in an in vivo model to clearly define the role of CD64-expressing cells in GBM recurrence.

In conclusion, although developing new drugs for treating GBM patients is challenging, multiomic analyses are a powerful tool to identify specific targets at various disease stages. Here, we identified genes and proteins that differ in their expression between the initial diagnosis and recurrence. Due to the tremendous heterogeneity of GBM tumors, we focused our attention on molecular players that promote recurrence, as they represent preferred targets for new therapies. The observation that CD64 and C1q-expressing cells strongly influence TTR at both gene and protein levels highlights them as potential targets. Our results suggest that CD64^+^ myeloid cells present in the GBM microenvironment could be a new focus for research aimed at better understanding resistance processes and preventing tumor relapse. We are currently developing an animal model able to mimic tumor recurrence, aiming to better understand the mechanisms involved in the regrowth of residual tumor cells. Targeting specific cell types, such as macrophages/microglia in genetically modified animals is currently under investigation.

## Supplementary Material

nlae108_Supplementary_Data
