# Supplementary material for: Multidimensional analysis of matched primary and recurrent glioblastoma identifies contributors to tumor recurrence influencing time to relapse
Source: J Neuropathol Exp Neurol. 2024 Oct 18;84(1):45–58. doi: 10.1093/jnen/nlae108 (PMC11659594; doi:10.1093/jnen/nlae108)

Figure S1

Barplots of the most differentially expressed genes in rGBM samples with STTR (**A**) and LTTR (**B**), and barplots and correlation scatterplots of these genes in STTR (**C**) and LTTR (**D**) samples selected from the GLASS Consortium database.

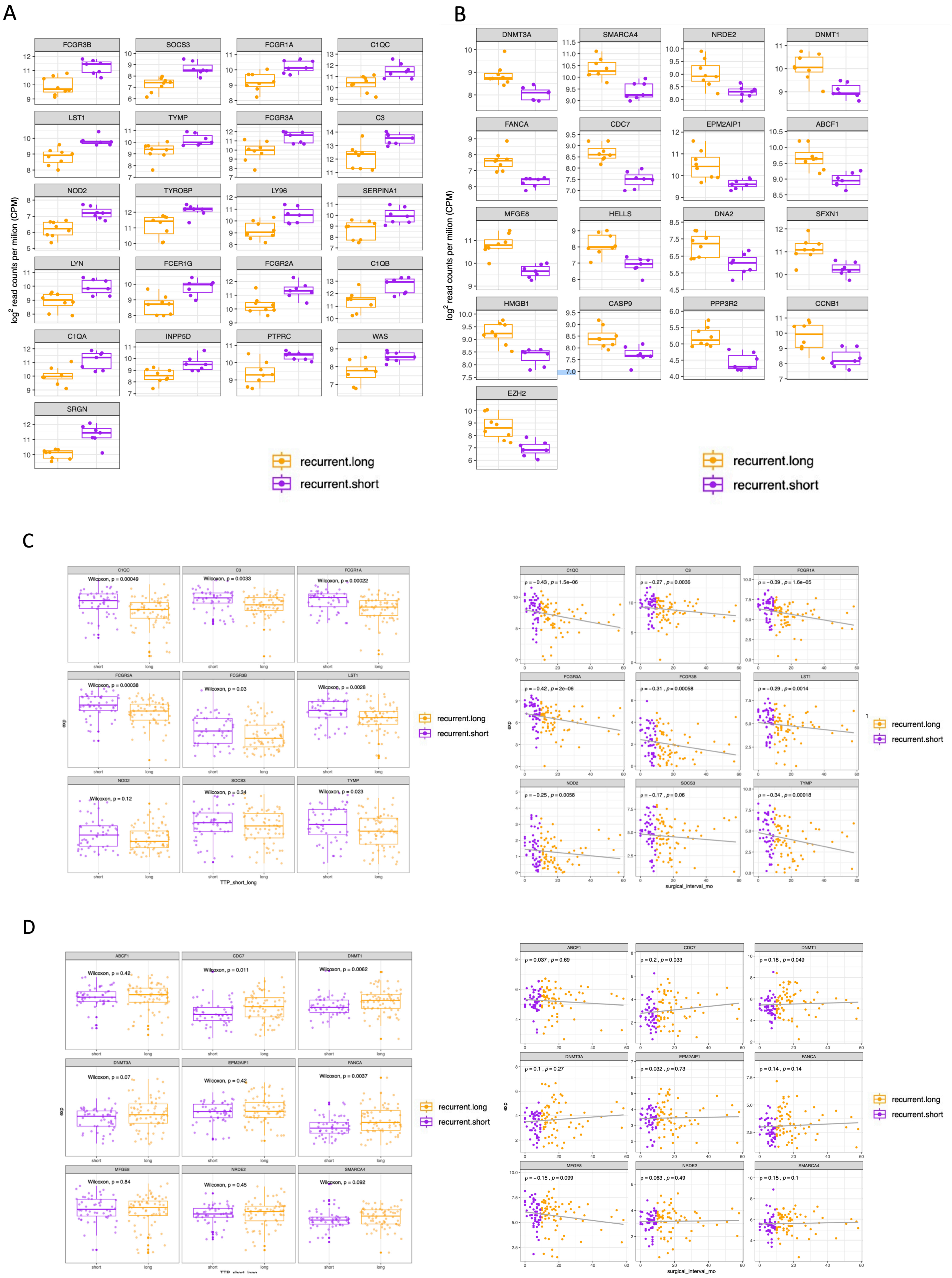

Supplement: nlae108_Supplementary_Data [file nlae108_supplementary_data.zip › nlae108_Supplementary_Data/figure S1 revision JNEN.pdf]
