# Supplementary material for: Multidimensional analysis of matched primary and recurrent glioblastoma identifies contributors to tumor recurrence influencing time to relapse
Source: J Neuropathol Exp Neurol. 2024 Oct 18;84(1):45–58. doi: 10.1093/jnen/nlae108 (PMC11659594; doi:10.1093/jnen/nlae108)

Figure S2
Boxplots (left panel) and scatter plots (right panel) of protein corresponding to the most differentially expressed genes between STTR and LTTR in rGBM samples. (**A**) enriched in STTR (**B**) enriched in LTTR.

A

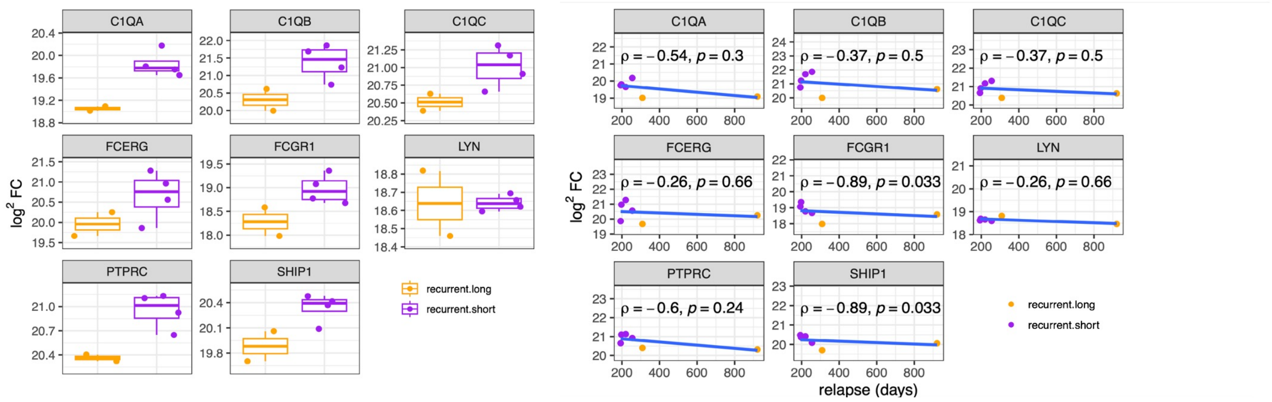

B

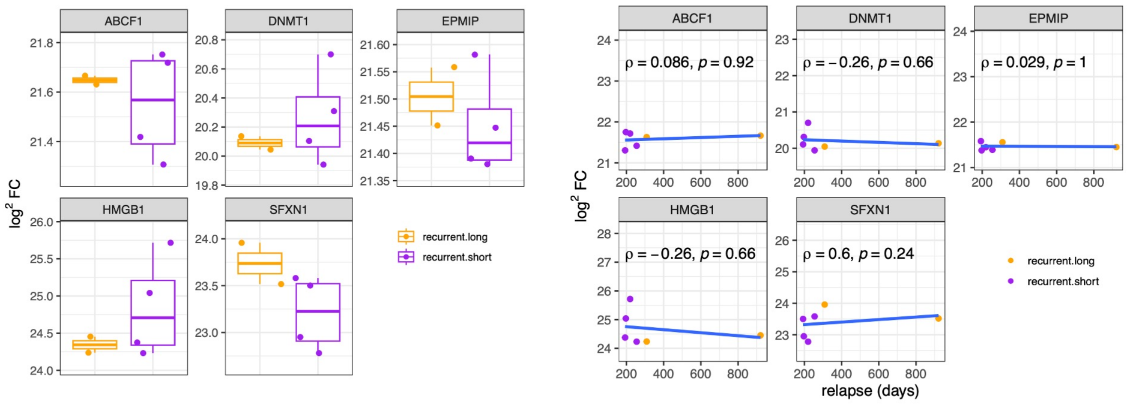

Supplement: nlae108_Supplementary_Data [file nlae108_supplementary_data.zip › nlae108_Supplementary_Data/figure S2 jnen revision.pdf]
