# Supplementary material for: Multidimensional analysis of matched primary and recurrent glioblastoma identifies contributors to tumor recurrence influencing time to relapse
Source: J Neuropathol Exp Neurol. 2024 Oct 18;84(1):45–58. doi: 10.1093/jnen/nlae108 (PMC11659594; doi:10.1093/jnen/nlae108)
Supplement: nlae108_Supplementary_Data [file nlae108_supplementary_data.zip › nlae108_Supplementary_Data/figure S3 revision JNEN.pdf]

Figure S3

Representative histological section of the TMA containing patient-paired pGBM and rGBM FFPE tissue cores and 2 liver tissue cores (**A**), and corresponding sample names (**B**).

A

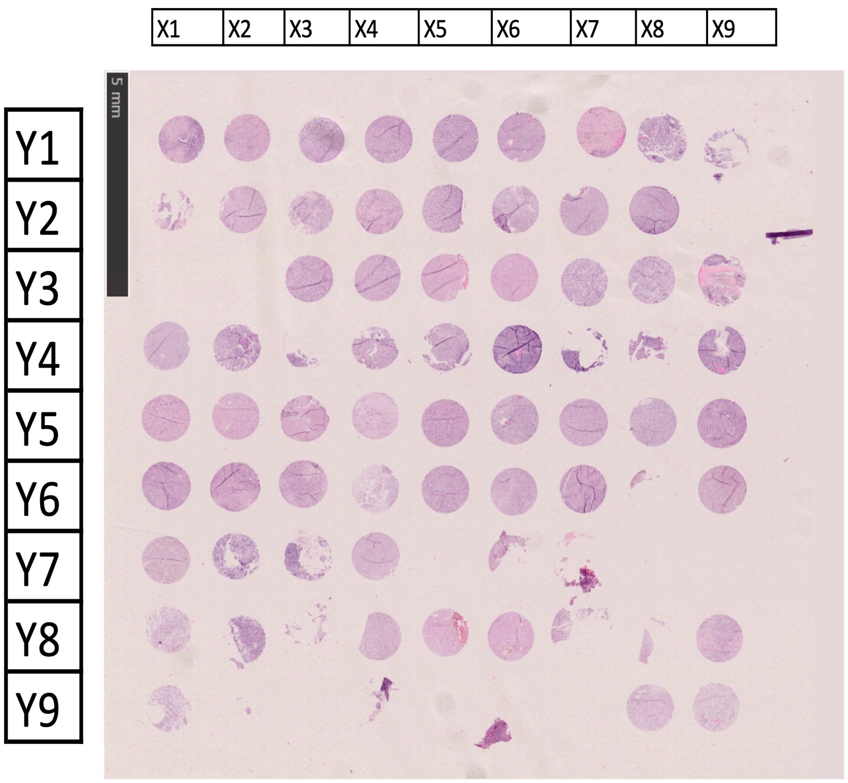

B

	X1	X2	X3	X4	X5	X6	X7	X8	X9
Y1	liver	liver	195P	195P	195P	195P	195P	195P	
Y2		356P	356P	356P	356P	282P	282P	282P	
Y3			182P	182P	182P	182P	185P	185P	185P
Y4	185P	185R		185R	185R	417P			417P
Y5	417R	417R	417R	417R	38P	38P	38P	38P	38R
Y6	38R	38R	38R	94P	94P	94P	94P		94R
Y7	94R	291P		291P		291R			
Y8	599R	599R	599R	599R	599P	599P			608P
Y9	608P			608R				182R	182R
