# Supplementary material for: Multidimensional analysis of matched primary and recurrent glioblastoma identifies contributors to tumor recurrence influencing time to relapse
Source: J Neuropathol Exp Neurol. 2024 Oct 18;84(1):45–58. doi: 10.1093/jnen/nlae108 (PMC11659594; doi:10.1093/jnen/nlae108)

Figure S4

Cell type abundances in counts (left) and as proportions (right) of CD64+ cell population (**A**) and GFAP+ cell populations (**B**) in individual ROIs from tumor samples.

A

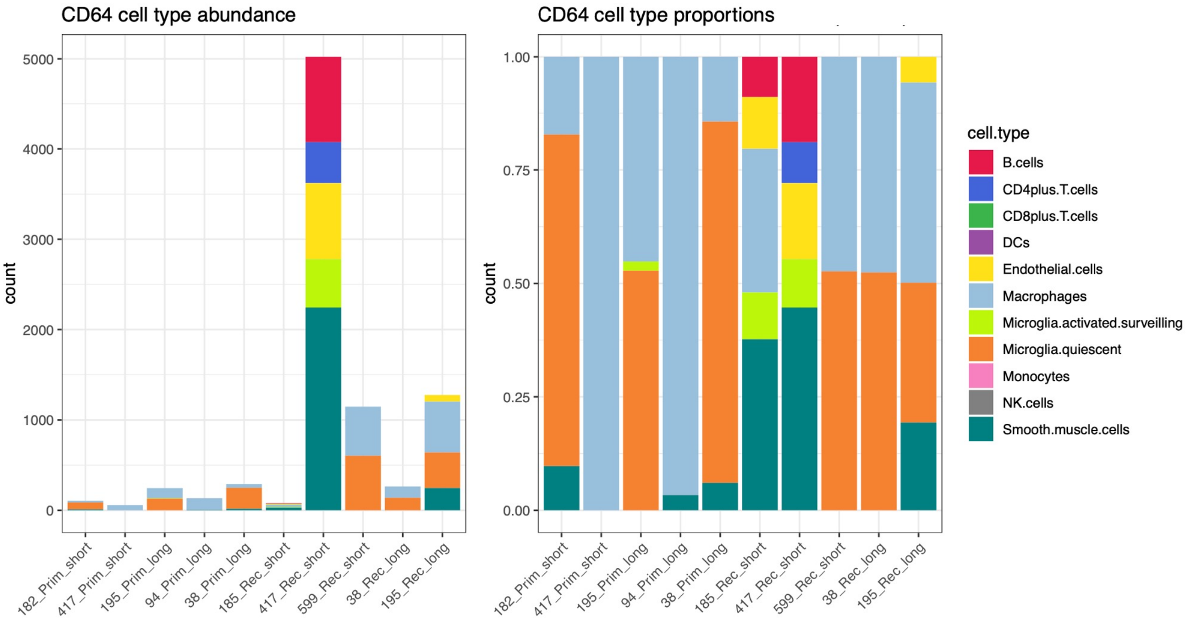

B

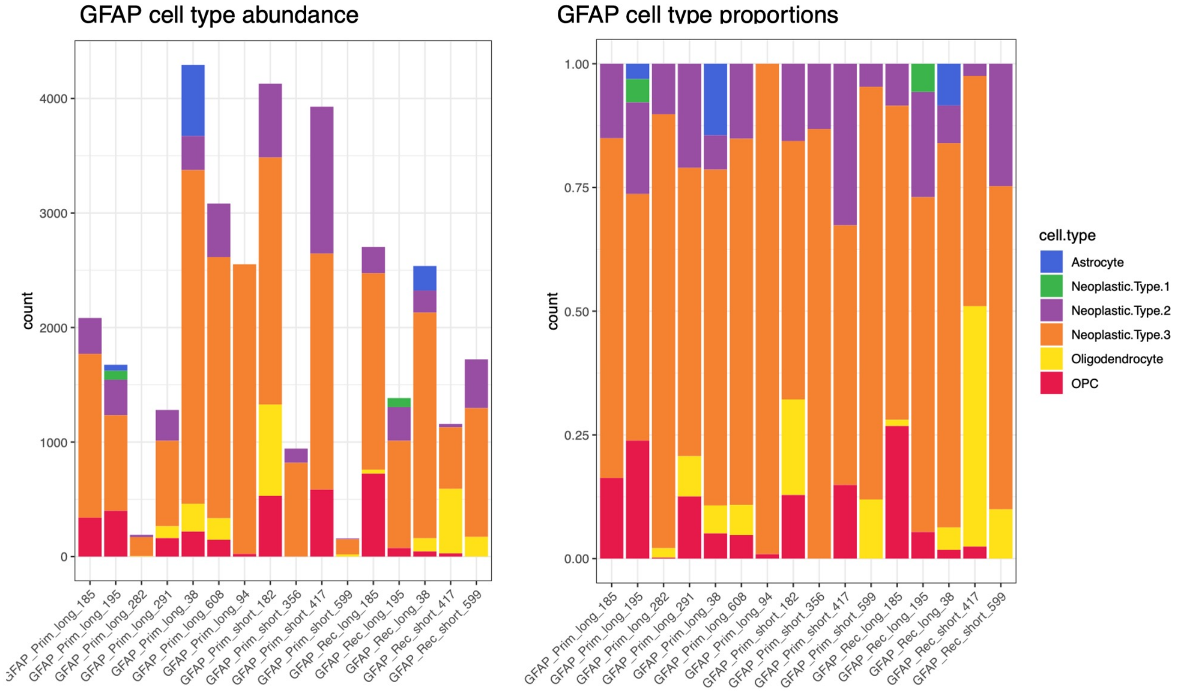

Supplement: nlae108_Supplementary_Data [file nlae108_supplementary_data.zip › nlae108_Supplementary_Data/Figure S4 revision JNEN.pdf]
