# Supplementary material for: Multidimensional analysis of matched primary and recurrent glioblastoma identifies contributors to tumor recurrence influencing time to relapse
Source: J Neuropathol Exp Neurol. 2024 Oct 18;84(1):45–58. doi: 10.1093/jnen/nlae108 (PMC11659594; doi:10.1093/jnen/nlae108)
Supplement: nlae108_Supplementary_Data [file nlae108_supplementary_data.zip › nlae108_Supplementary_Data/Supplementary Methods 1.docx]

**Supplementary Methods**

Protein extraction and digestion

After quality control a piece of 20mg of tissue was cut from the biopsy sample. The piece was then transferred into a microtube and grinded with a pellet pestle (Fisher Scientific, Reinach, Switzerland). Tissue was lysed in 8 molarity (M) Urea, 0.1M ammonium bicarbonate, phosphatase inhibitors (Sigma P5726&P0044) by sonication and proteins were digested as described previously (1), dried under vacuum and stored at -20°C until further use.

Tandem mass tag (TMT) proteomics

Sample aliquots comprising 10 μg of flow through peptides were labeled with isobaric tandem mass tags (TMTpro 16-plex, Thermo Fisher Scientific). Peptides were resuspended in 10 μl labeling buffer (2 M urea, 0.2 M HEPES, pH 8.3) by sonication and 2.5 μL of each TMT reagent was added to the individual peptide samples followed by a 1 h incubation at 25°C shaking at 500 rpm. After quenching of the labelling reaction, all samples were pooled and the pH was increased to 12 to remove TMT labels linked to peptide hydroxyl groups. The reaction was stopped by acidification. Finally, peptide samples were desalted using a C18 reverse-phase spin columns (Macrospin, Harvard Apparatus) according to the manufacturer’s instructions and dried under vacuum.

High performance liquid chromatography (HPLC) fractionation

TMT-labeled peptides were fractionated by high-pH reversed phase separation using a XBridge Peptide Ethylene Bridged Hybrid (BEH) C18 column (3,5 µm, 130 ångstrom (Å), 1 mm x 150 mm, Waters) on an Agilent 1260 Infinity HPLC system. Peptides were loaded in buffer A (20 mM ammonium formate in water, pH 10) and eluted using a two-step linear gradient from 2% to 10% in 5 minutes and then to 50% buffer B (20 mM ammonium formate in 90% acetonitrile, pH 10) over 55 minutes at a flow rate of 42 µl/min. Elution of peptides was monitored with an ultraviolet (UV) detector (215 nm, 254 nm) and a total of 36 fractions were collected, pooled into 12 fractions using a post-concatenation strategy as previously described (2) and dried under vacuum.

LC-MS/MS analysis

Dried peptides were resuspended in 0.1% aqueous formic acid (Buffer A) and subjected to LC–MS/MS analysis using a Q Exactive HF Mass Spectrometer fitted with an EASY-nLC 1000 (both Thermo Fisher Scientific) at 60°C. Peptides were resolved using a RP-HPLC column (75μm × 30cm) with C18 resin (ReproSil-Pur C18–AQ, 1.9 μm resin; Dr. Maisch GmbH) at a flow rate of 0.2 μLmin^-1^. The following gradient was used for peptide separation: from 5% solvent B (80% acetonitrile, 0.1% formic acid in water) to 15% solvent B over 10 min, to 30% solvent B over 60 min, to 45 % solvent B over 20 min, to 95% solvent B over 2 min, followed by 18 min at 95% solvent B.

The mass spectrometer was operated in DDA mode with a total cycle time of approximately 1 s. Each MS1 scan was followed by high-collision-dissociation (HCD) of the 10 most abundant precursor ions with dynamic exclusion set to 30 seconds.

The acquired raw-files were analysed using the SpectroMine software (Biognosis AG, Schlieren, Switzerland). Spectra were searched against a human database consisting of 20742 protein sequences (downloaded from Uniprot on 20190307) and 392 commonly observed contaminants. Raw reporter ions intensities of protein group specific PSMs were exported and used for quantification.

For each TMTpro 16-plex experiment, raw PSMs intensities were summed within a protein group and normalized using quantiles method (3). The normalization between multiple TMT experiments was performed as described here (4).

References:

1. Ahrné E, Glatter T, Viganò C, Schubert C, Nigg EA, Schmidt A. Evaluation and Improvement of Quantification Accuracy in Isobaric Mass Tag-Based Protein Quantification Experiments. J Proteome Res. 2016;15(8):2537-47.

2. Wang Y, Yang F, Gritsenko MA, Clauss T, Liu T, Shen Y, et al. Reversed-phase chromatography with multiple fraction concatenation strategy for proteome profiling of human MCF10A cells. Proteomics. 2011;11(10):2019-26.

3. Gatto L, Lilley KS. MSnbase-an R/Bioconductor package for isobaric tagged mass spectrometry data visualization, processing and quantitation. Bioinformatics. 2012;28(2):288-

4. Plubell DL, Wilmarth PA, Zhao Y, Fenton AM, Minnier J, Reddy AP, et al. Extended Multiplexing of Tandem Mass Tags (TMT) Labeling Reveals Age and High Fat Diet Specific Proteome Changes in Mouse Epididymal Adipose Tissue. Mol Cell Proteomics. 2017;16(5):873-90.
