# Supplementary material for: Multidimensional analysis of matched primary and recurrent glioblastoma identifies contributors to tumor recurrence influencing time to relapse
Source: J Neuropathol Exp Neurol. 2024 Oct 18;84(1):45–58. doi: 10.1093/jnen/nlae108 (PMC11659594; doi:10.1093/jnen/nlae108)
Supplement: nlae108_Supplementary_Data [file nlae108_supplementary_data.zip › nlae108_Supplementary_Data/Supplementary methods 2.docx]

**Supplementary methods 2**

Spatial transcriptomic

Slide-mounted FFPE TMA was processed for antigen retrieval using a heat induced epitope retrieval protocol for 20 minutes followed by a 5-min wash with 1μg/mL proteinase K solution. TMA was next incubated overnight with GeoMx RNA detection probes containing-photocleavable oligos. Next, slides were stained using a mixture of conjugated antibodies for identification of tissue morphology: anti-GFAP-594 (Novus Biologicals, NBP2-33184DL594; 1:1000) and anti-CD64 (purified with Abcam purification kit (ab102784) and conjugated with AlexaFluor647 (ab269235); 1:50) for the labeling of tumor cells and myeloid cells, respectively. In addition, Syto83 was used for DNA labeling (ThermoFisher Scientific, USA, 1:25). Stained slides were loaded onto a GeoMx instrument and scanned. Custom masks were created to define ROIs of interest for UV illumination. Once ROIs were defined, each area of interest was exposed to 385 nm light, releasing the indexed oligonucleotides which were collected and deposited in a 96-well plate for subsequent processing. Sequencing libraries were generated by PCR from the oligos and sequenced using Illumina NovoSeq according to the manufacturer’s protocol.
